# Phenotyping of Korean patients with better-than-expected efficacy of moderate-intensity statins using tensor factorization

**DOI:** 10.1371/journal.pone.0197518

**Published:** 2018-06-13

**Authors:** Jingyun Choi, Yejin Kim, Hun-Sung Kim, In Young Choi, Hwanjo Yu

**Affiliations:** 1 Department of Computer Science and Engineering, Pohang University of Science and Technology, Pohang, Republic of Korea; 2 Department of Medical Informatics, College of Medicine, The Catholic University of Korea, Seoul, Republic of Korea; University of Tampere, FINLAND

## Abstract

Several studies have been conducted to evaluate the efficacy of statins in Korean and Asian patients. However, most previous studies only observed the percent reduction in low-density lipoprotein cholesterol (LDL-C) and did not consider the effects of various patient conditions simultaneously, such as abnormal test results, patient demographics, and prescribed drugs before taking a statin. Moreover, the characteristics of the patients whose percent reduction in LDL-C was higher than expected were not provided. Therefore, in this study, we aimed to derive meaningful phenotypes by using tensor factorization to observe the characteristics of the patients whose percent reduction in LDL-C was higher than expected among patients taking moderate-intensity statins. In addition, we used the derived phenotypes to predict how much the LDL-C levels of new patients decreased. We consequently identified eight phenotypes that represented the characteristics of the patients whose percent reduction in LDL-C was higher than expected. Moreover, the latent representations of the derived phenotypes achieved prediction performance similar to that obtained using the raw data. These results demonstrate that the derived phenotypes and latent representations are useful tools for observing the characteristics of patients and predicting LDL-C levels. Additionally, our findings provide direction on how to conduct clinical studies in the future.

## 1 Introduction

One of the important factors for arteriosclerosis is low-density lipoprotein cholesterol (LDL-C) [1]. High levels of LDL-C cause arteriosclerosis. In addition, high LDL-C levels can increase the probability of developing cardiovascular diseases (CVDs), such as ischaemic heart diseases [2]. For this reason, patients are prescribed statins to reduce their LDL-C levels and help prevent CVDs [3–5].

Doctors can prescribe patients various statins with different dosages. The expected efficacy of statins depends on the types and dosages of the statins; thus, statins should be prescribed considering the target achievement rate. The most widely used statin guideline is the ACC/AHC guideline [6]. According to this guideline, rosuvastatin (10 mg) is classified as moderate-intensity statin therapy and is expected to reduce LDL-C levels by 30 to 50% [6].

However, this guideline is not suitable for Korean and Asian patients [7, 8] because it is based on studies that were conducted in limited Asian populations. For example, according to a previous study [8], some moderate-intensity statins are sufficient for lowering LDL-C levels in Korean patients. Although taking high-intensity statin therapy such as atorvastatin (40∼80 mg) and rosuvastatin (20∼40 mg) is recommended to reduce the LDL-C levels by more than 50 percent according to the ACC/AHC guideline [6], some moderate-intensity statins can replace these high-intensity statins in Korean patients with LDL-C levels ≥190 mg/dl [8]. Moreover, in previous studies [7, 9], statins were classified as high-intensity statins, moderate-to-high-intensity statins, moderate-to-low-intensity statins and low-intensity statins for Korean patients. However, the previous studies [7, 8] only evaluated the percent reduction in LDL-C after taking statins. The effects of various patient conditions, such as abnormal test results, patient demographics, and prescribed drugs before taking a statin, were not considered together. Moreover, the various characteristics of the patients whose percent reduction in LDL-C was higher than expected were not reported in previous studies [7, 8]. In practice, the percent reduction in LDL-C after taking statins can be higher than expected. For example, for a moderate-intensity statin that is known to lower LDL-C levels by up to 50% [6], LDL-C levels can occasionally decrease by more than 50%. These previous studies [7, 8] did not report the characteristics of the patients whose LDL-C levels decreased by more than 50%. To prescribe suitable statins and avoid drug overdoses or side effects [10–14], we need to conduct further research to determine the patient conditions that maximize the efficacy of statins.

In the real world, analyzing electronic health records (EHRs) is not easy because EHRs contain noisy, irregular, sparse data and do not map to the medical concepts used by clinical researchers. Therefore, we cannot easily extract meaningful medical concepts (i.e., phenotypes) from raw EHRs and must devote substantial time and effort to obtain these concepts. For this reason, clinical researchers and domain experts have recently used computational phenotyping, which is the process of automatically extracting phenotypes from EHR data using machine learning techniques such as deep learning methods [15, 16] and dimensionality reduction methods [17–25]. One of the various machine learning techniques for computational phenotyping is tensor factorization. Tensor factorization can take the multidimensional structure into account and capture complex interactions among high-dimensional EHR data. Due to this advantage, several applications based on tensor factorization have been proposed [17–25].

In this study, we aimed to derive meaningful phenotypes using tensor factorization to determine when the expected efficacy of some moderate-intensity statins was higher than expected among patients taking moderate-intensity statins. Additionally, we aimed to predict how much the LDL-C levels of new patients decrease using the derived phenotypes.

## 2 Materials and methods

Our data analysis procedure consisted of the following three steps: 1) data preprocessing from a hyperlipidemia dataset, 2) deriving phenotypes via tensor factorization, and 3) predicting the percent reduction in LDL-C.

### 2.1 Data preprocessing

For this study, we used a hyperlipidemia dataset from EHRs from Seoul St. Mary’s Hospital. The EHR data were collected between January 2009 and December 2015. This dataset contains several types of information, such as patient demographics, prescribed drugs before taking a statin, lab test results and prescribed statins. First, each patient who took one statin from among ten moderate-intensity statins was classified into one of six age groups (i.e., young men, middle-aged men, elderly men, young women, middle-aged women and elderly women) according to their age (age<40, 40≤age≤65, age>65) and gender (male, female). Second, we transformed drug prescriptions and lab test results into patient condition before taking a stain. We only considered the drug prescription histories within one year. For the lab tests, we preprocessed 6 test results at the first and second visits (45 to 225 days). We transformed abnormal test results to a binary type (0 for absence and 1 for presence). Glucose, HbA1C, high-density lipoprotein (HDL), LDL and total cholesterol (TC) were discretized into meaningful ranges. For example, a glucose level ≥126 mg/dl suggests that the patient has diabetes mellitus [26]. After preprocessing the diagnosed disease and lab test results, we obtained 19 patient conditions (13 abnormal test results and six drugs) before taking a stain. Then, we represented the transformed data as the number of co-occurrences between the age groups, the patient conditions and the statins of each patient. This co-occurrence is a natural representation for describing the interaction among an age group, patient condition and statin. The information of a patient was represented as a third-order tensor with age group, patient condition and statin modes. Finally, we constructed a fourth-order tensor with patient, age group, patient condition and statin modes for 2,235 patients. Each element of the tensor indicated whether a certain patient who belonged to an age group and had a patient condition had taken a statin. The percent reduction in LDL-C (△LDL-C) between the first and second visits was calculated as $100\%\times \frac{\left(first\;visit-second\;visit\right)}{first\;visit}$. According to the △LDL-C of patients, each patient was classified into the better-than-expected efficacy (△LDL-C ≥ 50%) group or the known efficacy (30% ≤ △LDL-C < 50%) group.

### 2.2 Generating phenotypes using tensor factorization

We used tensor factorization to derive meaningful phenotypes. Among the various tensor factorization methods, we used the nonnegative CANDECOMP–PARAFAC alternating Poisson regression (CP-APR) model [27], which is an extended CP model. CP-APR has stochastic constraints on the factor matrix; thus, we can easily interpret the elements of the factor matrix. Using CP-APR, we decomposed the constructed tensor X into *R* components, as follows: $X\approx \sum_{r=1}^{R}\lambda_{r}a_{r}\circ \;b_{r}\circ \;c_{r}\circ \;d_{r}$, where **a**_*r*_, **b**_*r*_, **c**_*r*_ and **d**_*r*_ are vectors; λ_*r*_ is a scalar; and ∘ is the outer product of vectors. Each component consists of λ_*r*_, **a**_*r*_, **b**_*r*_, **c**_*r*_ and **d**_*r*_.

We defined a phenotype as a set of age groups, patient conditions and associated statins that can occur together in a patient. **b**_*r*_ represents the age groups that are involved in the *r*-th phenotype. **c**_*r*_ represents how much the certain patient conditions before taking statins are involved in the *r*-th phenotype. **d**_*r*_ represents the certain statins that are involved in the *r*-th phenotype. For example, the *r*-th phenotype is defined using **b**_*r*_, **c**_*r*_ and **d**_*r*_. Using the phenotypes, each patient can be expressed as the latent representation of a patient, which consists of *R* values. λ_*r*_ **a**_*r*_ represents the degree to which the patients are involved in the *r*-th phenotype. Each element of λ_*r*_ **a**_*r*_ is the value of the latent representation for the *r*-th phenotype. Using the latent representations, we trained a binary logistic regression. Then, we categorized the phenotypes into the better-than-expected efficacy group and the known efficacy group according to the magnitudes of the coefficients in the binary logistic regression. We filtered out some phenotypes that were not statistically significant (i.e., *p*-values < 0.05 of the binary logistic regression). Details on computational phenotyping via CP-APR can be found in a previous study [17]. We used MATLAB software and the cp_apr function in MATLAB Tensor Toolbox Version 2.5 [28] from Sandia National Laboratories to represent tensors and to compute tensor operations.

### 2.3 Predicting low-density lipoprotein cholesterol levels

For new patients with moderate-intensity statins, we predicted whether their percent reduction in LDL-C was higher than expected. We used binary logistic regression as the prediction model, which was trained on the latent representations from the tensor factorization process.

To generate a new patient’s latent representation, we projected the new patient’s data $\tilde{X}$ onto the space of derived phenotypes. We calculated the new patient’s latent representations that best approximated $\tilde{X}\approx \sum_{r=1}^{R}\tilde{\lambda_{r}}\tilde{a_{r}}\circ \;b_{r}\circ \;c_{r}\circ \;d_{r}$. We performed the prediction with stratified 10-fold cross validation. We selected 90% of all patients as the training set and the remainder as the test set (10%) for each trial. To evaluate the prediction performance, we used accuracy, recall, precision, f-measure and the area under the curve (AUC) of the prediction model and reported the average results after ten repetitions.

### 2.4 Ethics

This study was approved by the Catholic University’s Institutional Review Board (IRB number: KC15EISI0103). The data from the participants were de-identified. All users provided written informed consent prior to participating in the study.

## 3 Results

### 3.1 Data preprocessing

We used the EHRs of 2,235 patients taking moderate-intensity statins from Seoul St. Mary’s Hospital in South Korea. Approximately 44.56% of the patients were classified into the better-than-expected efficacy group (Table 1).

**Table 1:** Demographic characteristics of the patients.

Age group	Better-than-expected efficacy group	Known efficacy group
	N (%)	N (%)
Elderly men	164 (16.46)	215 (17.35)
Elderly women	244 (24.50)	285 (23.00)
Middle-aged men	265 (26.61)	314 (25.34)
Middle-aged women	290 (29.12)	367 (29.62)
Young men	20 (2.01)	37 (2.99)
Young women	13 (1.30)	21 (1.70)
**Total**	996 (100.00)	1239 (100.00)

N = the number of patients.

The patients’ ages ranged from 19 to 100 years. There were 1,015 males (better-than-expected efficacy group = 449 and known group = 566) and 1,220 females (better-than-expected efficacy group = 547 and known group = 673). A total of 908 patients were in the elderly age group (better-than-expected efficacy group = 408 and known group = 500), and 1,236 participants were in the middle age group (better-than-expected efficacy group = 555 and known group = 681).

All patients took one statin from among 10 moderate-intensity statins (Table 2).

**Table 2:** Prescribed statins of the patients by LDL-C reduction group.

Statin therapy	Better-than-expected efficacy group	Known efficacy group
	N (%)	N (%)
Atorvastatin (10 mg)	209 (20.99)	441 (35.6)
Atorvastatin (20 mg)	72 (7.23)	57 (4.6)
Fluvastatin XL (80 mg)	2 (0.20)	16 (1.29)
Pitavastatin (2 mg)	99 (9.94)	225 (18.16)
Pitavastatin (4 mg)	8 (0.80)	14 (1.13)
Pravastatin (40 mg)	20 (2.01)	95 (7.67)
Rosuvastatin (10 mg)	500 (50.20)	284 (22.92)
Rosuvastatin (5 mg)	29 (2.91)	33 (2.66)
Simvastatin (20 mg)	56 (5.62)	72 (5.81)
Simvastatin (40 mg)	1 (0.10)	2 (0.16)
**Total**	996 (100.00)	1,239 (100.00)

N = the number of patients.

The most frequently prescribed statin was rosuvastatin (10 mg), which was prescribed for 35.08% of all patients (better-than-expected efficacy group = 500 and known group = 284). It was followed by atorvastatin (10 mg), 29.08%, and pitavastatin (2 mg), 14.50%.

In terms of HDL levels, 18.75% of patients had HDL-C levels of <40 mg/dl (Table 3). The percentages of patients who had LDL-C levels of 100∼129 mg/dl (near optimal), 130∼159 mg/dl (borderline high), 160∼189 mg/dl (high) and ≥190 mg/dl (very high) were 31.86%, 40.67%, 19.42% and 8.05%, respectively. The percentage of patients who had an abnormal TC level of 200∼239 mg/dl was 45.28%, whereas 30.02% of the patients had TC levels of ≥240 mg/dl. The most frequently prescribed drug before taking statins was thyroxine (7.29%).

**Table 3:** Clinical characteristics of the patients at baseline (the first visit).

Patient condition	Better-than-expected efficacy group	Known efficacy group
	N (%)	N (%)
AST(GOT) <14 or >20 U/L	584(58.63)	690(55.69)
Glucose 100∼125 mg/dl (prediabetes) [26]	249(25.00)	361(29.14)
Glucose ≥126 mg/dl (diabetes) [26]	429(43.07)	421(33.98)
HbA1C 6∼6.4% (prediabetes)	68(6.83)	141(11.38)
HbA1C ≥6.5% (diabetes)	473(47.49)	462(37.29)
HDL 40∼59 mg/dl [29]	588(59.04)	726(58.60)
HDL <40 mg/dl [29]	177(17.77)	242(19.53)
LDL 100∼129 mg/dl (near optimal) [29]	259(26.00)	453(36.56)
LDL 130∼159 mg/dl (borderline high) [29]	405(40.66)	504(40.68)
LDL 160∼189 mg/dl (high) [29]	217(21.79)	217(17.51)
LDL ≥190 mg/dl (very high) [29]	115(11.55)	65(5.25)
TC 200∼239 mg/dl (borderline high) [29]	428(42.97)	584(47.13)
TC ≥240 mg/dl [29]	360(36.14)	311(25.10)
2 Bisphosphonate	34(3.41)	38(3.07)
Fenofibrate	22(2.21)	24(1.94)
Omega-3	13(1.31)	12(0.97)
Propranolol	12(1.20)	17(1.37)
Thyroxine	63(6.33)	100(8.07)
Warfarin	5(0.50)	8(0.65)

N = the number of patients.

Overall, the tensor constructed for the analysis consisted of 9,877 non-zero values, and its size was 2,235 patients by 6 age groups by 19 patient conditions by 10 statins.

### 3.2 Generating phenotypes using tensor factorization

First, we derived 25 phenotypes from the training set by setting *R* = 25, and then we removed 14 phenotypes that were not statistically significant according to the *p*-values. According to the coefficient of the binary logistic regression, the selected phenotypes consisted of eight phenotypes of the better-than-expected efficacy group (phenotypes 2, 3, 5, 6, 11, 13, 15 and 17) and three phenotypes of the known efficacy group (phenotypes 14, 23 and 24) (Table 4).

**Table 4:** Selected phenotypes.

Phenotype	Coefficient	*p*-value	λ	Prevalence
2	0.2606	0.0000**	883.00	11.01%
3	0.2561	0.0000**	645.51	8.46%
5	0.2720	0.0000**	596.61	9.31%
6	0.2129	0.0002**	573.00	6.58%
11	0.1768	0.0239**	307.39	8.73%
13	0.1718	0.0226**	225.00	2.73%
14	-0.2476	0.0160**	221.00	2.60%
15	0.2030	0.0073**	217.00	2.37%
17	0.4113	0.0005**	203.09	6.85%
23	-0.2669	0.0394**	129.00	1.52%
24	-0.3742	0.0483**	82.33	3.98%

** *p* < 0.05.

The positive coefficient indicates that increasing the corresponding values of the latent representation increases the probability that the percent reduction in LDL-C is higher than expected (△LDL-C≥50%). Additionally, we calculated the prevalence indicating how many patients were relevant to the phenotype by counting the number of patients with a value of latent representation of the phenotype that was larger than zero and dividing by the total number of patients.

**Fig 1:**
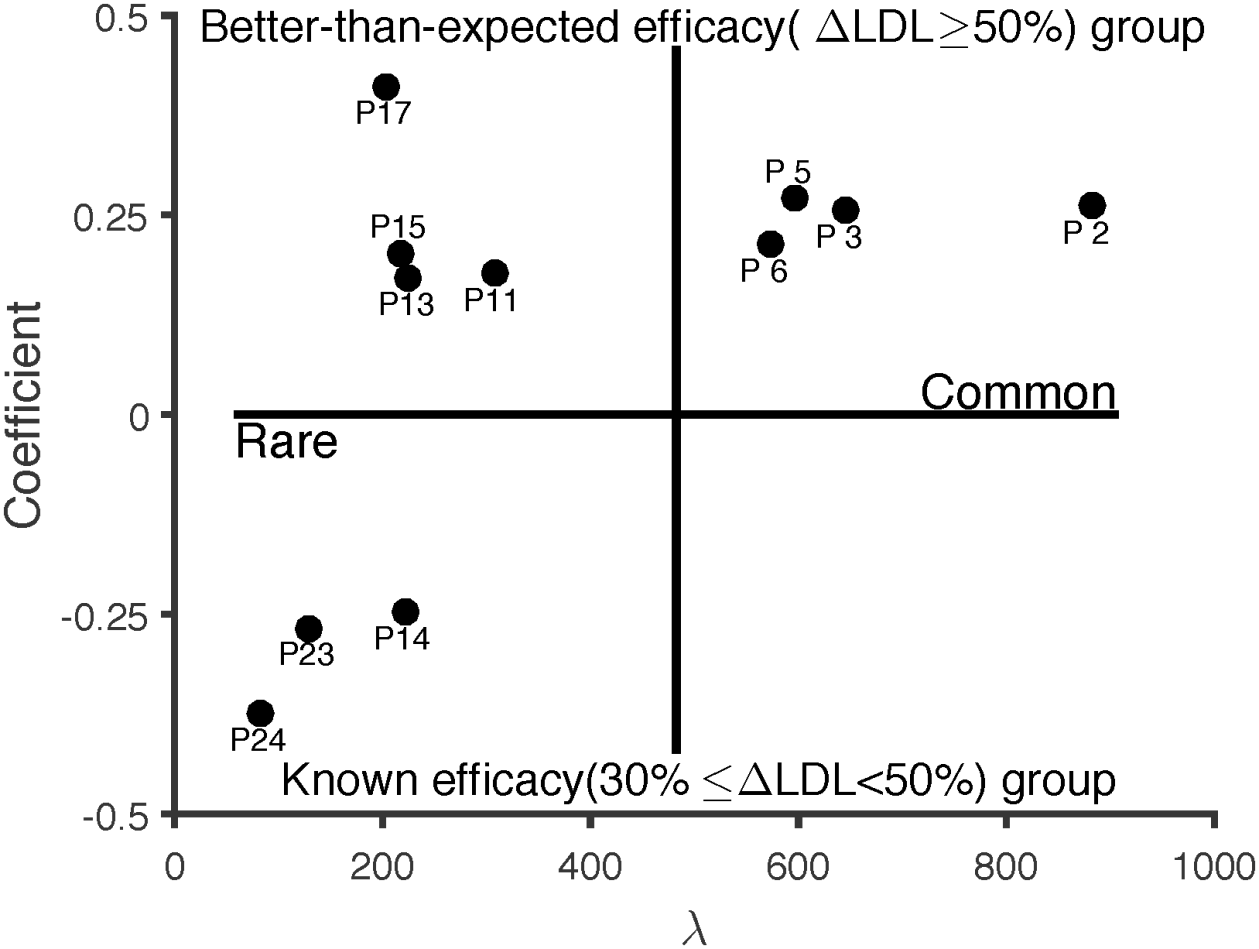
Phenotype map.

λ_*r*_ indicates how many co-occurrences of the *r*-th phenotype are observed in patients; thus, we also categorized them into common phenotypes or rare phenotypes according to λ (Fig 1).

The frequency (λ) of common phenotypes (phenotypes 2, 3, 5 and 6) was larger than the frequency (λ) of rare phenotypes (phenotypes 11, 13, 14, 15, 17, 23 and 24). To observe the characteristics of the better-than-expected efficacy group, we report the involvement of the age groups, patient conditions and associated statins in Table 5. For interpretability, we only considered the non-zero values of **b**_*r*_, **d**_*r*_ of more than 0.1 and **c**_*r*_ of more than 0.01. For lab tests, we reported the six most involved abnormal test results for each phenotype.

**Table 5:** Eight phenotypes of the better-than-expected efficacy group.

Phenotype	Statin	Age group	Patient condition		Proportion (N)
			Abnormal test result	Drug	
2	Rosuvastatin (10 mg) [96.1%]	middle-aged women [98.3%]	AST [13.7%]	Thyroxine [2.6%]	[10.60%] (237)
			HDL 40∼59 mg/dl [13.6%]		
			TC ≥240 mg/dl [13.4%]		
			TC 200∼239 mg/dl (borderline high) [8.6%]		
			LDL 130∼159 mg/dl (borderline high) [8.5%]		
			HbA1C ≥6.5% (diabetes) [7.8%]		
3	Rosuvastatin (10 mg) [100.0%]	elderly women [100.0%]	HDL 40∼59 mg/dl [16.6%]	Thyroxine [2.0%] Bisphosphonate [1.2%]	[7.34%] (164)
			AST [12.6%]		
			TC 200∼239 mg/dl (borderline high) [12.1%]		
			LDL 130∼159 mg/dl (borderline high) [11.6%]		
			HbA1C ≥6.5% (diabetes) [11.5%]		
			Glucose ≥126 mg/dl (diabetes) [9.8%]		
5	Rosuvastatin (10 mg) [100.0%]	middle-aged men [100.0%]	Glucose ≥126 mg/dl (diabetes) [15.8%]	Fenofibrate [1.3%]	[6.22%] (139)
			HbA1C ≥6.5% (diabetes) [15.8%]		
			TC 200∼239 mg/dl (borderline high) [13.6%]		
			AST [12.5%]		
			LDL 130∼159 mg/dl (borderline high) [12.1%]		
			HDL 40∼59 mg/dl [11.8%]		
6	Rosuvastatin (10 mg) [92.5%]	elderly men [90.2%]	AST [13.4%]	Fenofibrate [1.0%]	[6.44%] (144)
			HbA1C ≥6.5% (diabetes) [11.9%]		
			HDL 40∼59 mg/dl [10.8%]		
			Glucose ≥126 mg/dl (diabetes) [10.3%]		
			LDL 100∼129 mg/dl (near optimal) [9.8%]		
			TC 200∼239 mg/dl (borderline high) [9.6%]		
11	Rosuvastatin (10 mg) [100.0%]	middle-aged men [100.0%]	TC ≥240 mg/dl [20.2%]	Thyroxine [2.9%]	[3.18%] (71)
			HDL 40∼59 mg/dl [16.8%]		
			Glucose 100∼125 mg/dl (prediabetes) [15.9%]		
			LDL 160∼189 mg/dl (borderline high) [14.3%]		
			AST [13.9%]		
			HbA1C 6∼6.4% (prediabetes) [8.1%]		
13	Atorvastatin (20 mg) [53.3%] Simvastatin (20 mg) [37.3%]	elderly women [100.0%]	AST [13.3%]	Bisphosphonate [1.3%]	[2.73%] (61)
			Glucose ≥126 mg/dl (diabetes) [12.0%]		
			HbA1C ≥6.5% (diabetes) [11.1%]		
			HDL 40∼59 mg/dl [10.2%]		
			TC 200∼239 mg/dl (borderline high) [10.2%]		
			LDL 130∼159 mg/dl (borderline high) [9.3%]		
15	Atorvastatin (20 mg) [54.4%] Rosuvastatin (5 mg) [45.6%]	middle-aged women [100.0%]	HDL 40∼59 mg/dl [14.7%]	Thyroxine [1.4%]	[2.37%] (53)
			AST [12.0%]		
			HbA1C ≥6.5% (diabetes) [9.7%]		
			Glucose ≥126 mg/dl (diabetes) [9.2%]		
			TC 200∼239 mg/dl (borderline high) [9.2%]		
			TC ≥240 mg/dl [9.2%]		
17	Rosuvastatin (10 mg) [68.6%] Atorvastatin (10 mg) [31.4%]	elderly women [100.0%]	TC ≥240 mg/dl [45.3%]	Bisphosphonate [1.0%]	[1.92%] (43)
			LDL 160∼189 mg/dl (borderline high) [28.1%]		
			LDL ≥190 mg/dl (very high) [11.3%]		
			AST [9.3%]		
			Glucose 100∼125 mg/dl (prediabetes) [5.0%]		
Others					[59.19%] (1323)

N = the number of patients.

The proportion indicating how many patients have the maximum value of the latent representation of the phenotype was calculated by counting the number of patients with the maximum value of the latent representation of the phenotype and dividing by the total number of patients. In total, approximately 40.81% of the patients have the maximum value of the eight phenotypes of the better-than-expected efficacy group. Among them, six phenotypes (2, 3, 5, 6, 11 and 17) were involved in rosuvastatin (10 mg), and two phenotypes (13 and 15) were mainly involved in atorvastatin (20 mg).

### 3.3 Predicting low-density lipoprotein cholesterol levels

For new patients, we predicted whether their LDL-C levels decrease by more than 50% to demonstrate the effectiveness of the derived phenotypes and the latent representations. We compared our prediction results with the raw feature matrix with 2,235 × 35 columns, in which each row consisted of the various information of a patient (six age groups, 19 patient conditions and 10 statins were represented by a 1 × 35 vector). Table 6 provides the means and standard deviations of the prediction performance over stratified 10-fold cross validation.

**Table 6:** Prediction performance.

Data	AUC	Accuracy	Recall	Precision	F-measure
Raw data	0.6979±0.019	66.67±1.27	71.02±5.30	69.75±2.78	70.19±1.76
Latent representation	0.6872±0.018	67.70±1.69	76.19±2.99	68.93±1.94	72.33±1.49

Consequently, we observed that the latent representations of the derived phenotypes achieved prediction performance similar to that obtained using the raw data. The largest difference is 5.17% in terms of recall. The recall obtained by the latent representations was 76.19%, whereas the recall of the raw data was 71.02%.

## 4 Discussion

This study derived phenotypes from a hyperlipidemia dataset to observe when the expected efficacy of some moderate-intensity statins was higher than expected. Then, for new patient groups, we predicted whether their LDL-C levels decrease by more than 50% using the derived phenotypes and the latent representations. From the results of this study, we obtained the following conclusions.

First, we identified four common phenotypes (2, 3, 5 and 6) of the better-than-expected efficacy group according to λ. The common phenotypes represented patient groups consisting of male or female patients over the age of 40 that took rosuvastatin (10 mg) to decrease their LDL-C levels. In the patient groups, their patients had abnormal results of the HbA1C or glucose tests, which are used to screen for and diagnose diabetes [26] before taking a statin. HbA1C ≥6.5% or glucose ≥126 mg/dl suggests that the patients have diabetes mellitus [26]. More than 30% of all patients have the maximum value of the latent representation of these phenotypes. These findings were inconsistent with the ACC/AHC guideline [6]. Although patients took rosuvastatin (10 mg), which is known to reduce LDL-C levels by up to 50%, their LDL-C levels decreased by more than 50%. Therefore, these results are very valuable because patients can take a relatively low dose of rosuvastatin when they are highly associated with these phenotypes. According to the ACC/AHC guideline [6], high-intensity statin therapy such as atorvastatin (40∼80 mg) and rosuvastatin (20∼40 mg) is recommended to reduce the LDL-C level by more than 50%. However, patients who take high doses of statins may be more likely to experience side effects [10–14], such as kidney problems. Our results indicated that rosuvastatin (10 mg) is able to sufficiently reduce LDL-C levels and replace high-intensity statin therapy in Korean patients over the age of 40 with diabetes.

Second, we discovered four rare phenotypes (11, 13, 15 and 17) of the better-than-expected efficacy group. The rare phenotypes 11 and 17 correspond to patient groups in which patients had glucose 100∼125 mg/dl or HbA1C 6∼6.4%, which indicates that patients are more likely to develop prediabetes than are normal patients [26]. Middle-aged male patients in phenotype 11 took rosuvastatin (10 mg), whereas elderly female patients in phenotype 17 took rosuvastatin (10 mg) or atorvastatin (10 mg). In terms of the proportion, these phenotypes constitute 5.1% of the total patients. These phenotypes also represented the patients whose percent reduction in LDL-C is higher than expected. Unlike the previous phenotypes, rare phenotypes 13 and 15 represent patient groups in which female patients with diabetes mellitus (HbA1C ≥6.5% or glucose ≥126 mg/dL) over the age of 40 took atorvastatin (20 mg). Phenotypes 13 and 15 were also associated with simvastatin (20 mg) and rosuvastatin (5 mg), respectively. Approximately 5% of the patients have the maximum value of the latent representation of these phenotypes, and their LDL-C levels decrease by more than 50%.

Additionally, for new patients, we predicted whether their LDL-C levels decrease by more than 50% to observe the effectiveness of the derived phenotypes and the latent representations. According to the coefficient, increasing the corresponding values of the latent representation of the phenotypes of the better-than-expected efficacy group increased the probability that the percent reduction in LDL-C was higher than expected (△LDL-C≥50%). In terms of prediction performance, the latent representations of the derived phenotypes achieved prediction performance similar to that obtained using the raw data.

This study has some limitations. First, we only considered patients who achieved △LDL-C≥30% to determine when the expected efficacy of some moderate-intensity statins was higher than expected. Future research should be conducted with patients who achieved △LDL-C<30% to observe when the expected efficacy of some moderate-intensity statins was lower than expected. Second, we only evaluated moderate-intensity statins. In the future, additional studies need to be performed for high-intensity statins and low-intensity statins. Third, our findings are based on a retrospective data analysis from a hyperlipidemia dataset; thus, we cannot conclude that the various characteristics of the phenotypes only have an influence on reducing LDL-C levels. Therefore, prospective studies, such as a clinical study, must be conducted to determine the efficacy of statins with phenotypes.

Nevertheless, this study has important implications. The previous studies [7, 8] only showed the LDL-C reduction before and after taking statins and did not consider the effects of various patient characteristics, such as abnormal test results, patient demographics, and prescribed drugs. In our study, we showed that the derived phenotypes and latent representations are useful tools for observing the characteristics of patients who achieved a better-than-expected LDL-C reduction and predicting whether the prescribed statin can decrease the LDL-C level of a new patient by more than expected. Moreover, our findings provide direction on how to conduct clinical studies in the future. For example, rather than retrospective studies such as our study, we need to conduct a clinical study to verify that atorvastatin (20 mg) can reduce LDL-C levels by more than expected in the case of female patients over the age of 40 with diabetes mellitus. Then, doctors will be able to prescribe more suitable statins than at present and avoid both drug overdoses and side effects [10–14].

## 5 Conclusion

This study aimed to derive meaningful phenotypes from a hyperlipidemia dataset to observe when the expected efficacy of some moderate-intensity statins was higher than expected among patients with moderate-intensity statins. In addition, we aimed to predict how much the LDL-C levels of new patients decrease using the derived phenotypes. First, we represented the hyperlipidemia dataset as a fourth-order tensor with the number of co-occurrences between the age groups, the patient conditions and the statins of each patient. Using tensor factorization, we derived phenotypes from the constructed tensor. For new patients, we predicted whether their percent reduction in LDL-C was higher than expected. Consequently, we identified eight phenotypes of the better-than-expected efficacy group. Moreover, the latent representations of the derived phenotypes achieved prediction performance similar to that obtained using the raw data. These results demonstrate that the derived phenotypes and latent representations are useful tools for observing the characteristics of the better-than-expected efficacy group and predicting whether the LDL-C level decreases by more than expected. Additionally, our findings provide direction on how to conduct clinical studies in the future.
